# Editorial: Cognitive enhancement by brain stimulation techniques

**DOI:** 10.3389/fnhum.2026.1824005

**Published:** 2026-04-01

**Authors:** Elias Ebrahimzadeh, Hamid Soltanian-Zadeh

**Affiliations:** 1 CIPCE, School of Electrical and Computer Engineering, College of Engineering, University of Tehran, Tehran, Iran; 2 School of Cognitive Sciences, Institute for Research in Fundamental Sciences (IPM), Tehran, Iran; 3 Departments of Research Administration and Radiology, Henry Ford Health, Detroit, MI, United States

**Keywords:** brain stimulation, cognitive enhancement, cranial electrotherapy stimulation (CES), low-intensity pulsed ultrasound (LIPUS), repetitive transcranial magnetic stimulation (rTMS), transcranial alternating current stimulation (tACS), transcranial direct-current stimulation (tDCS)

## Introduction

Throughout history, humanity has persistently sought to augment its inherent capabilities, encompassing both cognitive and motor functions. Among the various tools explored for this purpose, brain stimulation techniques represent a domain whose potential has not yet been fully appreciated. Until recently, the application of these techniques was predominantly confined to rehabilitative contexts aimed at restoring lost functions. Consequently, the exploration of their capacity to enhance the cognitive abilities of healthy individuals—improving functions such as decision-making, working memory, attention, cognitive control, and learning—remained a relatively minor strand of literature, with findings often emerging as indirect byproducts of rehabilitation research. This Research Topic, therefore, is dedicated to a focused investigation of these very studies, seeking to evaluate the efficacy of brain stimulation techniques specifically for cognitive enhancement in healthy populations. By curating this Research Topic on “*Cognitive Enhancement by Brain Stimulation Techniques*,” our goal is to provide a comprehensive platform that examines the latest advancements in the field, fosters the sharing of critical insights among researchers, and offers a valuable resource for the broader scientific community. Ultimately, we aim to illuminate the significance of this area of inquiry and stimulate further research, paving the way for the development of more refined and effective methods for cognitive enhancement.

To advance this underexplored yet promising domain, this Research Topic was conceived to capture not only the latest empirical advancements but also the emerging perspectives and forward-looking insights that define the current landscape of cognitive enhancement through brain stimulation. We invited concise and forward-thinking contributions that highlight the state of the art while critically outlining the most pressing challenges and the innovative strategies required to address them. Rather than providing a retrospective overview of the past decade, the articles assembled here offer a snapshot of recent discoveries, novel developments, and evolving methodological approaches. A central thread running through these contributions is the effort to deepen our understanding of how brain stimulation techniques can modulate cognitive processes—such as decision-making, working memory, attention, and learning—in healthy individuals, and how these effects may be linked to underlying neural dynamics, including resting-state networks and cognitive processing pathways. By showcasing cutting-edge experimental methods and proposing future directions, this Research Topic seeks to inform, inspire, and guide researchers toward a more integrated and mechanistic understanding of cognition enhancement. We hope that the insights gathered here will not only illuminate the current potential of brain stimulation but also help chart a course for more targeted and effective interventions in the years to come.

## Research Topic coverage

The present Research Topic brings together a diverse body of work that reflects both the breadth and the evolving depth of research in cognitive enhancement via brain stimulation. Comprising nine contributions in total, the Research Topic includes three original research articles, two systematic reviews, two study protocols, one clinical trial, and one brief research report. Together, these papers offer a multidimensional perspective on the field, spanning empirical investigations, methodological advancements, and forward-looking frameworks. They collectively address fundamental questions regarding the mechanisms underlying stimulation-induced cognitive changes, while also highlighting innovative paradigms and emerging directions that are poised to shape future inquiry. Through this assemblage, we aim to provide readers with a representative overview of the current scientific landscape—one that captures not only where the field stands today but also the trajectories along which it is likely to evolve.

The study by Honma and Nomura, titled “*Gamma tACS Over the Prefrontal and Parietal Cortices Enhances Episodic Memory Performance*,” offers compelling evidence that gamma-frequency transcranial alternating current stimulation (tACS) can enhance episodic memory in healthy adults. Their finding that simultaneous stimulation of the prefrontal and parietal cortices improves memory discrimination at delayed time points underscores the promise of multi-site stimulation protocols for cognitive enhancement. By demonstrating sustained effects beyond the immediate stimulation period, this work advances our understanding of how noninvasive brain stimulation may modulate memory processes. These results open new avenues for developing targeted interventions to boost cognitive function in healthy populations.

In their brief research report, Baxendell et al. tested whether transcranial direct-current stimulation (tDCS) over the left dorsolateral prefrontal cortex could enhance positive mood induction effects in dysphoric individuals. Active tDCS reduced sadness at the final time point and led to less false recognition of negative words compared to sham. These preliminary findings suggest that combining brain stimulation with mood induction may reduce negative memory bias and promote more positive schema processing.

The study by Derakhshan et al. investigated the cognitive effects of repetitive transcranial magnetic stimulation (rTMS) in major depressive disorder, finding that while treatment successfully alleviated depressive symptoms, it did not significantly alter “hot” or “cold” cognitive functions. However, the research identified a specific pre-treatment cognitive profile—characterized by better sustained attention, facial expression recognition, and word recall—that predicted which patients would achieve remission. This work is significant as it moves beyond simply confirming rTMS's cognitive safety to highlighting potential cognitive biomarkers for treatment prognostication, paving the way for more personalized patient selection in depression care.

The study protocol by Cankaya et al. proposes a randomized controlled trial to evaluate tDCS in 120 patients with mild cognitive impairment (MCI) due to Alzheimer's or Parkinson's disease. The protocol uniquely compares stimulation targeting the dorsolateral prefrontal cortex vs. the lateral parietal cortex, a key node in the parietal memory network. Over 10 sessions, they will assess cognitive changes immediately and at 90-day follow-up, aiming to identify network-specific effects and move toward personalized stimulation approaches for early-stage neurodegeneration.

The study protocol by Xiong et al., titled “*Clinical study of wearable low-intensity pulsed ultrasound treatment in the improvement of executive function in obesity (the SLITE trial)*,” proposes a novel method using low-intensity pulsed ultrasound (LIPUS). In this double-blind randomized trial, they will apply wearable LIPUS to the dorsolateral prefrontal cortex of obese participants for 4 weeks to enhance executive function. The protocol combines this intervention with neuroimaging assessments like fMRI and fNIRS to explore underlying brain changes.

Biase et al. in their anodal tDCS study, investigated the causal roles of the left retro-splenial cortex (RSC) and hippocampus in spatial memory. They found that RSC stimulation selectively disrupted performance in room-centered trials with large viewpoint changes, providing causal evidence for its role in viewpoint-invariant spatial updating, while hippocampal stimulation yielded no reliable effects.

Okano et al. in their double-blind randomized clinical trial titled “*Effects of repeated cranial electrotherapy stimulation (CES) on physiological and behavioral responses to acute stress*,” investigated whether 20 sessions of CES could attenuate responses to an acute stressor. Despite robust stress induction, they found that active CES did not significantly differ from sham across physiological, biochemical, cognitive, or affective measures. These predominantly null findings challenge prevailing mechanistic accounts and suggest limited efficacy of CES for buffering acute stress in healthy individuals.

Providing moderate-quality evidence for the efficacy of non-invasive brain stimulation in DEACMP, a systematic review and meta-analysis by Ding et al. synthesized data from eight randomized controlled trials (RCTs) involving 607 patients. Their work, titled “*Effect of non-invasive brain stimulation on cognitive function and activities of daily living in patients with carbon monoxide poisoning*,” demonstrated significant improvements in both cognitive function and activities of daily living following NIBS interventions. Notably, the therapeutic effects were moderated by key factors including patient age, the duration of intervention, and the specific cortical site targeted, suggesting that personalized parameters may optimize outcomes. These findings offer valuable clinical guidance for tailoring neurorehabilitation strategies in this patient population.

Wang et al.s' systematic review and meta-analysis, titled “*Effects of multi-site non-invasive brain stimulation on cognitive impairment after stroke*,” synthesized six RCTs with 416 patients. The review demonstrates that multi-site non-invasive brain stimulation (NIBS) is superior to single-site stimulation for improving global cognitive function in stroke patients, particularly through multi-site TMS or combined TMS-tDCS protocols. These findings highlight the therapeutic potential of targeting distributed brain networks to enhance cognitive recovery after stroke.

## Conclusion

Collectively, the nine articles assembled in this Research Topic offer a multifaceted snapshot of the current state and future promise of cognitive enhancement through brain stimulation. The contributions span a wide methodological spectrum, from original research providing novel behavioral evidence—such as the episodic memory improvements following gamma-tACS reported by Honma and Nomura—to rigorous systematic reviews and meta-analyses that synthesize evidence across clinical populations. For instance, Ding and colleagues demonstrated the potential of NIBS in ameliorating cognitive deficits after carbon monoxide poisoning, while Wang et al. provided compelling evidence for the superiority of multi-site over single-site stimulation in post-stroke cognitive impairment. Alongside these, clinical trials like that of Okano et al. offered critical null findings that challenge existing assumptions about CES efficacy, underscoring the importance of rigorous, well-controlled studies. Protocol papers, such as the one by Xiong et al., further highlight ongoing efforts to unravel the causal roles of specific brain regions in cognition. Together, these works not only advance our understanding of how non-invasive brain stimulation can modulate cognitive functions in both healthy and clinical populations but also illuminate the key challenges and future directions for the field. They reinforce the notion that while the potential of brain stimulation for cognitive enhancement is substantial, realizing this potential will depend on continued methodological refinement, larger-scale trials, and a deeper integration of mechanistic insights. We hope this Research Topic will serve as both a valuable resource and a catalyst for further innovation in this rapidly evolving field.

